# Efficacious and safe orotracheal intubation for laboratory mice using slim torqueable guidewire-based technique: comparisons between a modified and a conventional method

**DOI:** 10.1186/s12871-016-0173-6

**Published:** 2016-01-18

**Authors:** Chieh-Shou Su, Hui-Chin Lai, Chih-Yen Wang, Wen-Lieng Lee, Kuo-Yang Wang, Ya-Ling Yang, Li-Chun Wang, Chia-Ning Liu, Tsun-Jui Liu

**Affiliations:** 1 Cardiovascular Center and Department of Anesthesiology, Taichung Veterans General Hospital, Taichung, Taiwan; 2 Institute of Clinical Medicine, National Yang Ming University, Taipei, Taiwan; 3 Departments of Medicine and Surgery, National Yang-Ming University School of Medicine, Taipei, Taiwan; 4 Department of Medicine, Chung-Shan Medical University School of Medicine, Taichung, Taiwan; 5 Department of Medicine, China Medical University School of Medicine, Taichung, Taiwan

**Keywords:** Mouse, Laboratory, Endotracheal intubation, Anesthesia

## Abstract

**Background:**

Tracheal intubation of laboratory mice remains essential yet challenging for most researchers. The aim of this study was to investigate whether this procedure can be more efficiently and safely accomplished by a novel method using slim and torqueable guidewires to guide access to the trachea.

**Methods:**

This study was carried out in an animal laboratory affiliated to a tertiary medical center. Mice weighing 22 to 28 g were subjected to various open-chest experiments after being anesthetized with intraperitoneal ketamine (100 mg/kg) and lidocaine hydrochloride (10 mg/kg). The oropharyngeal cavity was opened with angled tissue forceps, and the trachea was transilluminated using an external light. The vocal cords were then crossed using either the Conventional method with a 38-mm-long, end-blunted stiff needle as a guide for insertion of a 22-gauge, 25-mm-long intravenous catheter into the trachea, or the Modified method utilizing using a 0.014-inch-thin torqueable wire as the guide to introduce an identical tube over it into the trachea. The epithelial integrity of the trachea was later examined histologically when the animals were sacrificed either immediately after the surgery or at 28 days post-surgery, depending on the corresponding research protocols.

**Results:**

Orotracheal intubation was successfully completed in all mice using either the Conventional (*N* = 42) or the Modified method (*N* = 50). With the Modified method, intubation took less time (1.73 vs. 2.17 min, Modified vs. Conventional, *p* < 0.001) and fewer attempts (1.0 vs. 1.33, *p* < 0.001), and there were fewer procedural difficulties (0 % vs. 16.7 %, *p* = 0.009) and complications (0 % vs. 11.9 %, *p* = 0.041) compared with the Conventional method. Histological analysis revealed a significantly lower incidence of immediate (0 % vs. 39 %, *p* < 0.001) and late (0 % vs. 58 %, *p* < 0.001) injuries to the tracheal epithelial lining with the Modified method compared to the Conventional method.

**Conclusions:**

Tracheal intubation for laboratory mice can be completed efficiently, safely and atraumatically using the proposed Modified method employing readily available inexpensive instruments.

## Background

Mice are among the most commonly used laboratory animals, especially in the era of genetic modification [1, 2]. Tracheal intubation of these animals for subsequent experiments is indispensable for various in vivo research purposes [3–5], however it remains challenging due to the unfavorable anatomical characteristics of these animals including a small body, large incisors, tight mandible, anteriorly placed glottis, small larynx, narrow trachea, and rapidly mobile vocal cords [6, 7], all of which complicate the intubation procedure and lead to oral bleeding, pharynx edema, laryngeal perforation and esophageal mispositioning with conventional techniques. [8] The two critical steps to determine the success and safety of orotracheal intubation for mice are therefore clear visualization of the oropharygolaryngeal anatomy and accurate crossing of the vocal cords by the endotracheal tube. Some modified methods have been developed to overcome these difficulties, including the introduction of a first-going guide wire [6, 9, 10] into the tracheal orifice followed by advancement of an endotracheal tube over this wire, and the employment of devices such as a modified arthroscope with an affiliated light source and video camera [6, 9] or an optical fiber cable [11, 12] to provide clear and direct visualization of the entrance of the trachea. Most of these methods, however, require extensive training, mastery of complex techniques and the use of expensive devices [5, 6, 9, 11–13], and they still carry the risk of animal mortality from procedural failure.

As anesthesiologists and interventional cardiologists, we previously developed a secure means of performing orotracheal intubation in rats [3]. The aim of the present study was to examine whether the even tougher intubation of laboratory mice can be similarly efficaciously and safely performed using a simple method with a slim, soft-tipped, torqueable guide wire of the type commonly employed in human coronary interventional procedures. The results of this study document the feasibility and safety of this novel tracheal intubation technique for mice, and imply this method as a good option for scientists conducting this procedure for relevant research purposes.

## Methods

### Animals

Six- to eight-week-old C57B/6 mice weighing 22–28 g were obtained from the Taiwan National Laboratory Animal Center for our other research protocols involving open-chest surgery (murine models of cardiac ischemia-reperfusion [14, 15], acute/chronic myocardial infarction [16], or left ventricle pressure overload induced by transverse aortic constriction [17]). The animals were housed under controlled conditions (25 °C constant temperature, 55 % relative humidity, 12-h light/dark cycle) and fed with a standard pelleted diet and water ad libitum until subjected to the experiments. All animal procedures and experimental protocols were approved by the Institutional Animal Care and Use Committee (IACUC) of Taichung Veterans General Hospital (protocol number: La-97571; IACUC Chairman: Jaw-Ji Tsai; date of approval: 08-01-2009) and were carried out in accordance with the Guiding Principles of the American Physiological Society for the Care and Use of Animals in Research and Teaching.

### Materials

**Fig. 1:**
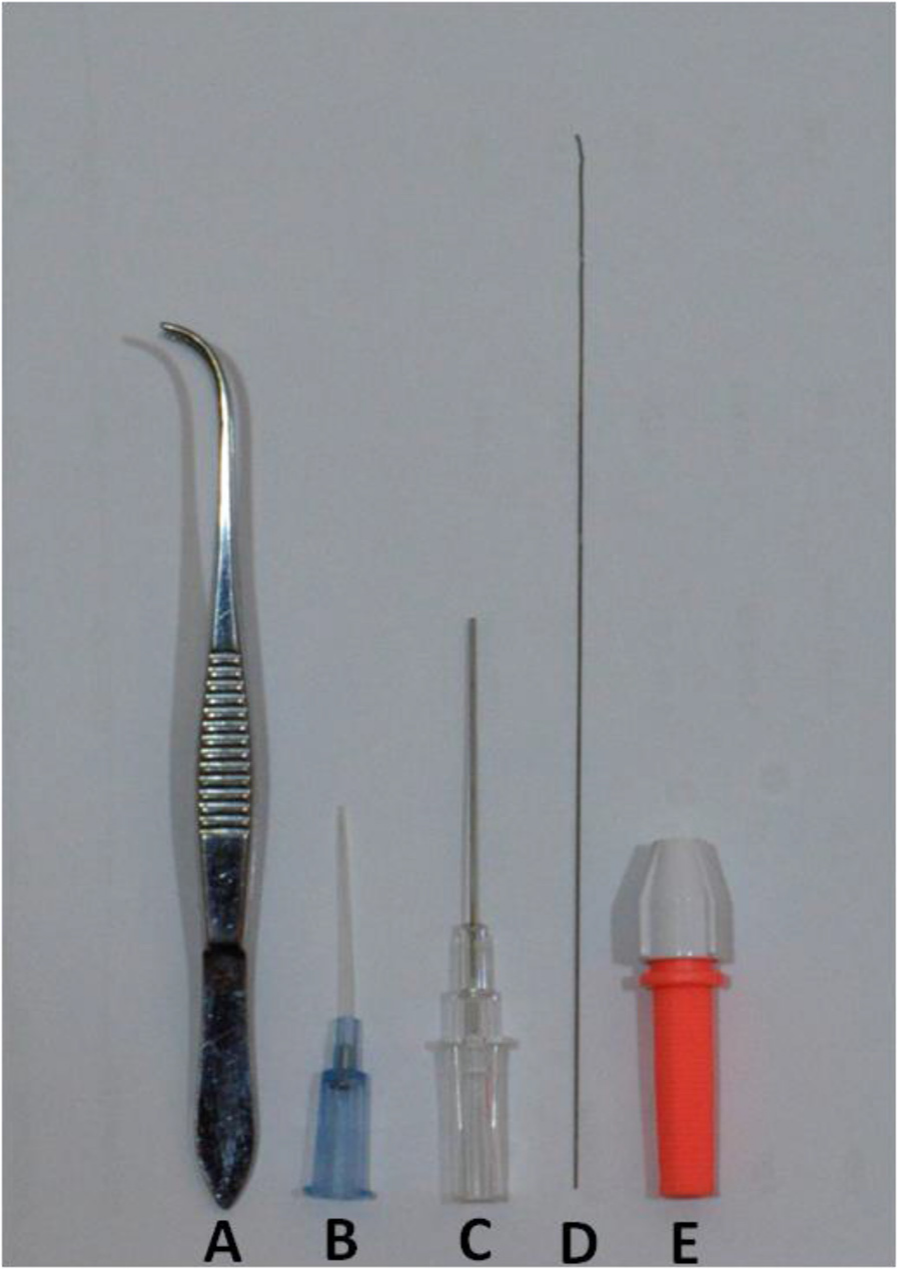
Equipment for the orotracheal intubation of laboratory mice. **a** tissue forceps acting as a laryngeal blade. **b** 22-G intravenous catheter. **c** 38-mm long, sharp tip-blunted needle stylet with a 30-mm hub. **d** 0.014-inch (0.36 mm) guide wire. **e** Guide wire-manipulating torque device

A specially designed plastic platform (30 × 20 cm) was attached to the head mount of a tripod head and fixed on a stand. The platform angle could be freely adjusted and then fixed to fit the user’s needs, as previously described [3]. Regular, straight, atraumatic smooth forceps (forceps 1) (GRAEFE 18-880-10, DANNORITZER Medizintechnik GmbH & Co. KG) were used to pull the tongue laterally as it would be in clinical and other research procedures. Tissue forceps (angled, length 100 mm) (forceps 2) (GRAEFE 18-883-10, DANNORITZER Medizintechnik GmbH & Co. KG) were used to open the mouth similar to the way a laryngoscope is used in humans (Fig. 1a). A 22-G intravenous catheter 25 mm in length with an internal diameter of 0.6 mm and a hub of 20 mm (Terumo Europe N.V., Leuven, Belgium) was used as the tracheal intubation tube (Fig. 1b). The associated needle stylet (38 mm long, 0.024 inch (0.61 mm) in diameter, connected to a 30-mm hub) with 2 mm of the most distal sharp tip removed (Fig. 1c), or a soft-tipped, 0.014-inch (0.36 mm)-wide coronary artery guide wire (Balance Middleweight Elite, Abbott Vascular, International BVBA, Belgium) which was cut to 12 cm from the default length of 190 cm (Fig. 1d), served to guide the tracheal tube into the trachea in both methods, as described below. A torque device (Radifocus ® Torque device, Terumo Europe N.V., Leuven, Belgium) was used to mechanically lock the tail of the guidewire (at around 4 cm from the tail end of the guide wire) to manipulate it across the vocal cords whenever needed (Fig. 1e). A 100 W halogen light source (MORITEX MHF-D100LR) was used to transilluminate the oropharynx from the ventral side of the mouse’s neck to facilitate localization of the trachea during intubation.

### Orotracheal intubation

**Fig. 2:**
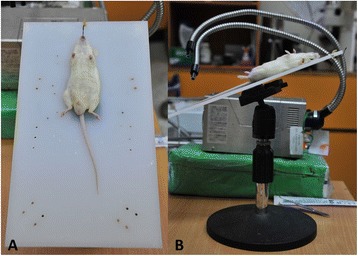
Animal placement for tracheal intubation. Panel **a**, the mouse was placed in the dorsal recumbent position on a specially-designed platform (30 × 20 cm) with the upper incisors hooked and fixed by a silk band on the proximal end of the platform. Panel **b**, the platform angle was adjusted to an angle of 45-60° to help straighten the curvature between the trachea and the larynx to enable clear visualization of the oropharyngeal cavity and facilitate tracheal intubation

**Fig. 3:**
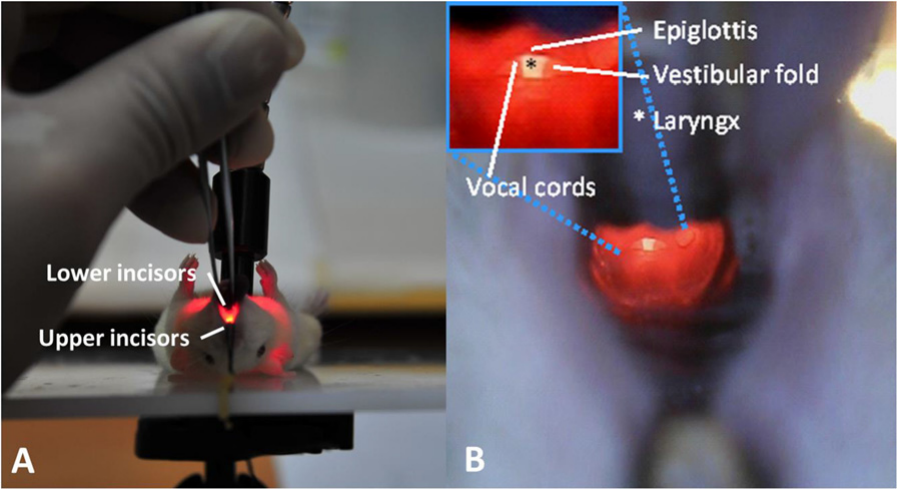
Illustration of a mouse’s oral cavity from the operator’s viewpoint. Panel **a**, the mouth was opened using angulated tissue forceps (forceps 2) and the tongue was pulled laterally with smooth forceps (forceps 1). A 100-W halogen light source with a flexible fiber-optic arm was aimed at the ventral side of the mouse’s neck to illuminate the trachea. Panel **b**, the oral cavity and the vocal cords were clearly transilluminated by the 100-W halogen light source. Left upper diagram, magnification of the laryngopharynx showing details of the orolaryngopharyngeal apparatus

All of the mice were anesthetized with a mixture of ketamine (100 mg/kg) and lidocaine hydrochloride (10 mg/kg) intraperitoneally, and then placed in the dorsal recumbent position on a specially designed platform with the upper incisors hooked and fixed with a silk band on the proximal end of the platform (Fig. 2, Panel a). The platform was then elevated to an angle of 45–60° to help straighten the curvature between the trachea and larynx (Fig. 2, Panel b) to allow for clear visualization of the oropharyngeal cavity. A 100 W halogen light source with a flexible fiber-optic arm was aimed at the ventral side of the mouse’s neck to elucidate the trachea just below the vocal cords (Fig. 3, Panel a). The mouth of the animal was opened with the regular, straight, atraumatic smooth forceps (forceps 1) and the tongue was pulled laterally. The angled, 10-cm long tissue forceps (forceps 2, working as a laryngoscope blade) were then inserted into the oral cavity along the tongue and then adjusted anterosuperiorly to displace the soft palate and to lift the epiglottis in order to clearly illustrate the vocal cords and trachea. The position of the fiber-optic arm of the light source was adjusted for each mouse to provide optimal illumination of the vocal cords and trachea (Fig. 3, Panel b). Orotracheal intubation was then performed using either the Conventional or Modified method as described below. For the mice intubated with the Conventional method (Conventional Group), the trachea was intubated directly with a 22-G intravenous catheter over a 38-mm stiff, straight, tip-blunted needle. For the mice intubated with the Modified method (Modified Group), a 0.014-inch coronary artery guide wire (Fig. 1d) controlled with a torque device (Fig. 1e) was initially inserted into the trachea. After removal of the torque device, a 22-G catheter was advanced into the trachea over the guide wire. After these intubation procedures, the catheter hub was connected to a rodent ventilator (ventilator, NEMI Scientific Inc., USA) delivering 120 breaths per minute at a volume of 200 μl [2]. Correct tracheal positioning of the catheter was confirmed by the rapid rise and fall of bilateral chest walls.

### Assessment of feasibility and safety

The technical feasibility of both methods for tracheal intubation was evaluated by the time taken to complete the intubation procedure (defined as the time from opening the animal’s mouth with the tissue forceps, advancing the endotracheal tube into the trachea to connection of the tube hub to the mechanical ventilator), the number of attempts needed, the difficulties encountered during the procedure (vigorous gag reflex interrupting intubation, excess salivary secretion hindering clear visualization of the larynx, and unusual resistance precluding smooth advancement of the tube into the trachea), and the overall success rate. Technical safety was assessed by the number of instances of esophageal mispositioning, massive oral bleeding, and procedure-related mortality. Tissue integrity was assessed by direct histological analysis of the intubated trachea in both the short and the long term. Short-term analysis was conducted in the mice subjected to open-chest surgical procedures with immediate sacrifice of the animal after completion of the experimental steps (cardiac ischemia-reperfusion injury and acute myocardial infarction models). In these mice, the hearts were explanted, the chest cavities opened and the tracheas exposed. The position of the tip of the tracheal tube was determined using an inverted microscope at 5x magnification. The tracheas were then harvested and their integrity between the vocal cords and the catheter tip position was examined histologically by a researcher blinded to group allocation, using H&E staining to determine whether the epithelium was damaged. Long-term analysis was performed in the mice that had recovered from anesthesia (chronic myocardial infarction and left ventricle pressure overload induced by transverse aortic constriction models). They were extubated with spontaneous breathing, returned to their cages and given standard care until they were sacrificed at 4 weeks for research purposes. In these animals, the integrity of the entire trachea from the vocal cords to bifurcation was histologically examined.

### Statistical analysis

Continuous variables are expressed as mean or median ± SD, and categorical variables are expressed as frequencies and percentages. Normally distributed continuous data were compared between groups using an unpaired Student’s t-test, and non-parametric continuous data were compared using the Mann–Whitney U test. Categorical variables were analyzed using the chi-square test with Yate’s correction. Statistical significance was defined as a *p* value < 0.05. All analyses were performed using SPSS software version 10.1 (SPSS, Inc., Chicago, IL, USA).

## Results

### Animals and outcomes of the tracheal intubation procedures

Each tracheal intubation (for both methods) was performed by a single researcher (a senior interventional cardiologist) on two respective mice as a learning exercise to define the feasibility of the methods and avoid inter-operator technical discrepancies. The Conventional intubation technique was used in 42 consecutive mice (Conventional Group), and the Modified technique was used in 50 consecutive mice (Modified Group). The characteristics of the animals and the consequences of the orotracheal intubation procedures are summarized in Table 1. All of the animals survived orotracheal intubation, underwent subsequent open-chest surgery without limb cyanosis or air leakage from the lungs, and were subjected to the short- and long-term experiments. With both methods, the tracheal orifice of all mice could be clearly visualized. However, the Modified method was associated with a shorter procedure time and only a single attempt needed to complete the intubation procedure in all 50 animals without causing intra-esophageal mispositioning, oral cavity bleeding or any other major complications compared with the Conventional method (Table 1).

**Table 1:** Baseline data and procedural characteristics of orotracheal intubation in both groups of mice

	Conventional group	Modified group	*P* value
Number, n	42	50	-
Body weight (g)	25.40 ± 1.50	25.24 ± 1.51	0.602
Endotracheal intubation time (minutes)	2.17 ± 0.26	1.73 ± 0.18	<0.001
Intubation attempt(s)	1.33 ± 0.6	1 ± 0	0.001
Success rate (%)	42 (100)	50 (100)	NS
Difficulties met during intubation			
Overall (%)	7 (16.7)^a^	0 (0)	0.009
Vigorous gag reflex (%)	4 (9.5)	0 (0)	0.086
Salivary secretion (%)	5 (11.9)	0 (0)	0.041
Resistance on advancement (%)	3 (7.1)	0 (0)	0.183
Complications			
Overall (%)	5 (11.9)	0 (0)	0.041
Esophageal disposition (%)	4 (9.5)	0 (0)	0.086
Oral bleeding (%)	1 (2.4)	0 (0)	0.457
Death (%)	0 (0)	0 (0)	NS

^a^More than one kind of difficulty/complication occurred during intubation in some mice

When performed by another two inexperienced researchers, only two practice procedures under the instruction of the senior interventional cardiologist of either the Conventional or Modified method were needed to achieve a success rate of 100 % within one attempt in the following 20 mice. The time required to complete tracheal intubation using the Conventional method by the inexperienced researchers was 2.11 ± 0.25 min, compared to 1.74 ± 0.28 min for the Modified method (*P* < 0.001, Modified vs. Conventional method). The body weights of the mice in the Conventional and Modified groups for the inexperienced researchers were 25.80 ± 1.19 g and 26.13 ± 1.23 g, respectively (*P* = 0.397). The body weights and intubation times for the mice in the Modified Group for the inexperienced researchers were comparable to those of the senior interventional cardiologist (t-test *P* = 0.142 and *P* = 0.921, body weight and intubation time, respectively).

### Histological assessment of the intubated tracheas

**Fig. 4:**
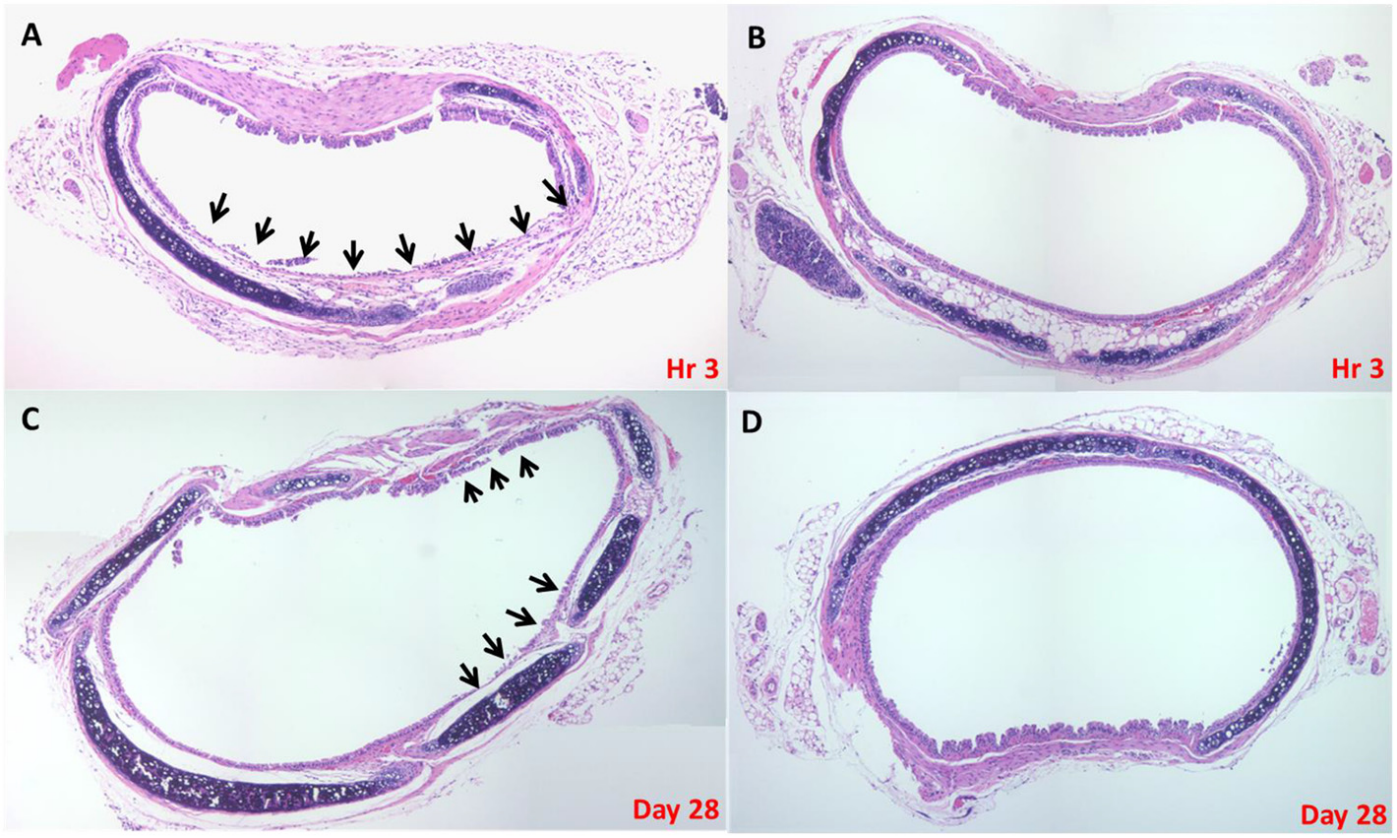
Histological examination (H&E stain) of the intubated trachea at the site around the tip of the tracheal tube in mice sacrificed immediately (average 3 h) after completion of the experiment (Panels **a** & **b**) or at 28 days after completion of a follow-up period (Panels **c** & **d**). **a** & **c**, representative tracheal tissue of mice in the Conventional group. The epithelial lining was focally interrupted and the associated cilia were lost (arrows). **b** & **d**, representative tracheal tissue of mice in the Modified group. The ciliated epithelium was intact and free from micro-damage

During necropsy, the tip of the tracheal tube was accurately located 2–3 mm from the tracheal carina in all animals in both groups without any identifiable gross tracheal bleeding. Microscopic examination of the tracheal segments harvested from the animals sacrificed shortly after open-chest surgery indicated that the epithelial lining near the tip of the tracheal tube was focally interrupted and the cilia damaged in 7 of 18 (39 %) mice in the Conventional Group (Fig. 4, Panel a), but was intact in all 22 animals in the Modified Group (*P* < 0.001) (Fig. 4, Panel b). Similar focal interruption of the epithelial lining and damage of the cilia were noted histologically in tracheal segments of 14 of 24 (58 %) mice of the Conventional Group (Fig. 4, Panel c) that were subjected to long-term follow-up, but not in any of the 28 animals in the Modified Group (*P* < 0.001) (Fig. 4, Panel d).

## Discussion

Tracheal intubation of laboratory mice before proceeding to open-chest surgery remains an essential yet challenging procedure for investigators due to anatomical characteristics that are not favorable for basic research [3–5]. The present study demonstrates that this difficult and time-consuming procedure can be quickly and safely completed using a novel method employing easily acquired and inexpensive tools including a halogen light, tissue forceps, a 0.014-inch guide wire and a 22-G IV catheter, without causing any short- or long-term complications. Heightened ethical concerns for animals used in research have focused on the preservation of life as the pivotal issue [18, 19], and the unique value of genetically modified mice underscores the need to protect them from inadvertent harm during experimental processes [20]. Our results demonstrate that the crucial yet potentially life-threatening tracheal intubation procedure can be performed more efficaciously and safely without the loss of animal life, thus suggesting the application of this novel method in laboratories requiring intubation of mice for subsequent experiments.

### Comparison of our orotracheal intubation methods with other methods

Orotracheal intubation of laboratory mice was first reported by Ho and Furst in 1973 [21]. Since then, a number of modifications in the procedure and instruments used have been proposed to improve the success rate and to reduce unnecessary loss of animal life. However, these reports have either not provided adequate details for researchers who want to replicate the protocols [22], or have indicated that the procedure can only be performed with complex and expensive equipment [6, 9, 11, 12] such as fiber-optic arthroscopes and endoscopes, or requiring operators with extensive training (three students and one physician needed to accomplish 20 consecutive intubations successfully within 60 s before performing subsequent intubations) [9]. Importantly, only a few studies have reported overcoming the two key difficulties in tracheal intubation, i.e., clear visualization of the tracheal orifice [9, 12, 23] and easy advancement of the endotracheal tube into the trachea. [6, 9, 10] Tools meant to fully open the oral cavity such as a metal laryngoscope blade [13] or a custom-made laryngoscope [5] have the disadvantages of obscuring visualization of the vocal cords and triggering pharyngeal-laryngeal reflexes, which can easily cause esophageal disposition and laryngeal injury. Instruments designed to reveal the location of the tracheal entrance such as expensive endoscopic instruments [11, 12, 24] including a modified arthroscope with affiliated light source plus video camera [6, 9] and an optic-fiber cable [11, 12] can easily induce a visual mismatch between the video images and the real oral cavity, thus needing a much longer learning curve to master.

On the other hand, for both of our methods, the mice were placed in the dorsal recumbent position at a 45-60° tilt with the upper incisors fixed by a silk band, thereby maximizing the opening of the oral cavity and aligning the operator’s line of sight with the epiglottis. Additional expansion of the operative field by tissue forceps to lift the epiglottis anterosuperiorly along with transillumination of the trachea from the ventral side of the mouse’s neck by a light source further helped localize the vocal cords and trachea visually. The tissue forceps used in this study (forceps 2) functioned as a miniature human laryngoscope with the curvature of its arms similar to the blade of the laryngoscope, and it also provided good view to allow successful endotracheal intubation. Importantly, they were slim enough not to obscure localization of the vocal cords and trachea, thus avoiding inadvertent esophageal intubation during advancement of the endotracheal tube. Our methods do not need expensive devices or extensive training, as with most of the other methods. Most importantly, the instruments we used in the Modified method to facilitate tracheal tube advancement, i.e., the floppy, slippery, thin guide wire (only 0.36 mm in diameter) and a torque device, could be delicately manipulated atraumatically to wherever the operator intended it to go, just as in a human coronary artery which is smaller than the trachea of a mouse. In fact, utilization of a guide wire to facilitate orotracheal intubation in mice has been described previously [6, 9, 10, 23], including Vergari et al [6, 9] who used a 0.4-mm guide wire, Hamacher et al [10] who used a 2F (0.67 mm in diameter) guide wire, and Thomas et al [23] who also used a straight 2F (0.67 mm in diameter) guide wire in their studies. The main difference between their methods and ours is that the guide wires we used in our Modified Group were smaller in diameter (only 0.36 mm) and designed specifically for human coronary artery interventions, which can be well controlled by a torque device to smoothly and accurately enter the trachea in the same manner as they are manipulated in a coronary artery without causing injury to the endothelium they touch. The results of the present study demonstrate the efficacy and safety of this novel modified procedure for the orotracheal intubation of laboratory mice. This tool is at least not inferior to other devices used for the same purpose, as reflected by the 100 % procedural success rate and no immediate or late complications.

### Comparison between the Modified and Conventional methods of tracheal intubation

Orotracheal intubation could be completed in all of our mice with either of our Conventional or Modified method, however the relatively longer time and greater number of attempts needed, along with significantly more difficulties encountered with the Conventional method indicate that the 0.024-inch (0.61-mm) stiff stylet used in the Conventional method was a less efficient instrument than the 0.014-inch (0.36 mm) floppy guide wire used in the Modified method as an introducer for the tracheal tube. The significantly higher incidence rates of procedure-related complications and short- and long-term tracheal tissue damage further suggests the potential harm that can be caused by a stiff stylet made from a needle with the end blunted. In contrast, the better performance of the 0.014-inch guide wire used in the Modified method in terms of ease of intubation and freedom from procedure-related complications or tracheal injury indicates that it is both an efficacious and atraumatic method to enter the extremely narrow tracheal opening and cross the rapidly vibrating vocal cords without triggering vigorous gag/salivary reflexes or damaging the tracheal epithelial lining. In addition, the torque device used for precise human percutaneous coronary interventions enhances the maneuverability of the guide wire to accurately enter the trachea, thus reducing esophageal disposition. Moreover, for experiments in which animals are kept alive for a period of time or are intubated repeatedly [11] such as for radiological imaging [24, 25], pulmonary ventilation or agonist challenging studies [1, 13, 26], the absence of short- and long-term tracheal injuries in the Modified method can the primary experiments, thus making it a more suitable method than others for a wider range of in vivo research.

In summary, tracheal intubation of laboratory mice for subsequent experiments can be accomplished quickly, safely and reliably with our proposed novel method employing a slanted platform, an external light source, tissue forceps, a slim and soft 0.014-inch guide wire, a torque device, and a modified tracheal tube, all of which can be readily acquired at low cost and easily assembled in a typical animal laboratory dedicated to cardiovascular research. This method can be considered as a good choice for relevant experiments, especially for those in which repeated tracheal intubation is necessary [26].

## Conclusion

Tracheal intubation of anesthetized mice undergoing experimental surgical procedures can be rapidly and successfully accomplished with the proposed novel technique using inexpensive readily available instruments including a slim, soft-tipped guide wire and an associated torque device without immediate or late complications. This technique can be considered the method of choice for this procedure, enabling researchers to quickly and safely establish a secure airway in mice for subsequent experimental procedures.
